# Rheumatoid Arthritis Increases the Risk of Nontuberculosis Mycobacterial Disease and Active Pulmonary Tuberculosis

**DOI:** 10.1371/journal.pone.0110922

**Published:** 2014-10-22

**Authors:** Jun-Jun Yeh, Yu-Chiao Wang, Fung-Chang Sung, Chia-Hung Kao

**Affiliations:** 1 Department of Internal Medicine and Family Medicine, Ditmanson Medical Foundation Chia-Yi Christian Hospital, Chiayi, Taiwan; 2 Chia Nan University of Pharmacy and Science, Tainan, Taiwan; 3 Meiho University, Pingtung, Taiwan; 4 Management Office for Health Data, China Medical University Hospital, Taichung, Taiwan; 5 College of Medicine, China Medical University, Taichung, Taiwan; 6 Graduate Institute of Clinical Medical Science, China Medical University College of Medicine, Taichung, Taiwan; 7 Department of Nuclear Medicine and PET Center, China Medical University Hospital, Taichung, Taiwan; Cambridge University, United Kingdom

## Abstract

**Background:**

Few studies have examined the association of rheumatoid arthritis (RA) with nontuberculosis mycobacterium (NTM) disease and pulmonary tuberculosis (PTB).

**Methods:**

We identified 29 131 patients with RA from the catastrophic illness registry who were diagnosed from 1998–2008; 116 524 patients without RA from inpatient data files were randomly frequency matched according to sex, age, and index year and used as a comparison group. Both groups were followed-up until the end of 2010 to measure the incidence of NTM disease and active PTB. We analyzed the risk of NTM disease and active PTB using the Cox proportional hazards regression models, controlling for sex, age, and Charlson comorbidity index (CCI).

**Results:**

The incidence of NTM disease was 4.22 times greater in the RA group than in the non-RA group (1.91 vs 0.45 per 10,000 person-years). The incidence of PTB was 2.99 times greater in the RA group than in the non-RA group (25.3 vs 8.46 per 10,000 person-years). After adjusting for age, sex, and CCI, the adjusted hazard ratios (HRs) of NTM disease and active PTB for the RA group were 4.17 (95% CI = 2.61–6.65) and 2.87 (95% CI = 2.55–3.23), respectively, compared with the non-RA group. In the first 2 years of follow-up, the RA group yielded corresponding adjusted HRs of 4.98 and 3.39 compared with the non-RA group. The follow-up time-specific RA group to the non-RA group HR of both the NTM disease and active PTB varied.

**Conclusion:**

This study can serve as a reference for clinical physicians to increase awareness regarding the detection of NTM disease and active PTB in RA patients among the any stage of the clinical course even without CCI.

## Introduction

Rheumatoid arthritis (RA) is the most common connective tissue disease (CTD) and exerts an increasing burden on health resources globally [1], [2]. Pulmonary infection is related to RA [3]. The reactivation of mycobacterium tuberculosis (MTB) infection is a major complication in patients treated with antitumor necrosis factor (anti-TNF) agents in RA [4]. Treating opportunistic infections emerging in RA is crucial, as is providing therapy for the original disease. MTB is a pathogen among the main opportunistic infections [5], but no study has observed that RA increases the risk of active pulmonary tuberculosis (PTB).

The chronic use of methotrexate (MTX) to treat RA can result in opportunistic infections such as pulmonary nontuberculosis mycobacterium (NTM) disease [6]. One study observed NTM disease in immunosuppressed RA patients using anti-TNF-alpha therapy [7]. Yamakawa et al showed disease deterioration because of an antirheumatic drug received during NTM disease follow-up [8]. However, no study has focused on whether RA increases the risk of NTM disease.

The Charlson comorbidity index (CCI) was originally developed to create a single-value summary for several comorbid conditions for breast cancer patients in 1984, and is suitable for assessing general medical inpatient populations [9],[10]. Among the comorbidity scales developed for general medical patients, CCI is the most popular and the easiest to apply [11]. Tiippana-Kinnunen et al observed that comorbidities increased during 15 years of RA, and patients with a high baseline CCI showed high disease activity, both in early disease and at the end-point [12].

PTB appears to increase in RA patients, independent of treatment [13], [14], although a U.S. study differed in this regard [15]. Furthermore, no research has discussed conditions comorbid with RA that are associated with NTM disease and active PTB. Therefore, we conducted a nationwide RA-cohort study to investigate whether RA increases the risk of NTM disease and active PTB. This is the first study to examine the association of RA and CCI with the development of NTM disease and active PTB among the clinical course by using time-dependent covariates in a general Asian population.

## Materials and Methods

### Data Source

The Taiwan National Health Insurance (NHI) is a universal insurance program established by the Taiwan Department of Health in March 1995 by consolidating 13 insurance programs. The insurance system has a coverage rate of 99% for 23.74 million people. The Taiwan NHI Research Database (NHIRD) (http://nhird.nhri.org.tw/en/index.htm) is authorized to provide insured registration files and original reimbursement claims data in the 1996–2010 periods.

In this study, we used two datasets included the all of the hospitalization clams data and the Registry for Catastrophic Illness Patient Database (RCIPD) of Taiwan, in which the International Classification of Diseases, 9th Revision, Clinical Modification (ICD-9-CM) was used to define diseases. To protect patient privacy, all personal identification numbers are encrypted before electronic files are released. Both datasets were scrambled with surrogate identifications of the insured and analyzed anonymously.

### Data Availability Statement

All data and related metadata are deposited in an appropriate public repository. The study population's data which were from Taiwan NHIRD (http://w3.nhri.org.tw/nhird//date_01.html) are maintained by Taiwan NHIRD (http://nhird.nhri.org.tw/). The National Health Research Institutes (NHRI) is a non-profit foundation established by the government.

### Ethics Statement

The NHIRD encrypts patient personal information to protect privacy and provides researchers with anonymous identification numbers associated with relevant claim information, including patients' sex, dates of birth, medical services utilized, and prescriptions. Patient consent is not required for accessing the NHIRD. This study was approved by the Institutional Review Board of China Medical University (CMU-REC-101-012). Our IRB specifically waived the consent requirement.

### Study Population

This study is a retrospective population-based cohort study. In Taiwan, the rheumatologists can apply for the catastrophic illness card for any patient with RA, who fulfils the presence of 4 or more diagnostic criteria based on the 1987 ACR criteria [16]. The application of the catastrophic illness card should be scrutinized by peer review. The patients with RA with a catastrophic illness card can be exempt from paying a copayment. We used the Registry for Catastrophic Illness Patient Database (RCIPD) to identify RA (ICD-9-CM 714) patients in the claims data, and the first-time RA diagnosis served as the index date from 1998 to 2008. Meanwhile, these consensuses of chest physician, infection specialist and personnel working in the coding room confirmed the new diagnosis of NTM disease and active PTB. The definition of NTM disease [17] and active PTB based on World Health Organization (W.H.O) report [18] and the American Thoracic Society (A.T.S) [17], [19], [20]. Furthermore, the Center for Disease Control (C.D.C) is the supervisor of the infection control of active PTB.These procedure may avoid bias such as misclassification.

The comparison group was 4 times the size of the RA group and consisted of patients without RA, selected randomly from the inpatient data files, frequency matched by age, sex, and diagnosis year. Patients with a history of NTM disease (ICD-9-CM 031.0, 031.2, 031.8, 0.31.9) or active PTB (ICD-9-CM 011) at the baseline, and those with incomplete age or sex information, were excluded. The follow-up person-years were calculated for each participant since index date until the diagnosis of new event (NTM disease or active PTB), the end of 2010, or withdrawal from the insurance system. Patients who had experienced NTM disease or active PTB prior to the baseline year (1996–1997) were excluded.

According to each participant's inpatient diagnosis, we calculated the CCI scores as the comorbidity measure. A weight was assigned in each indicated diagnosis and added together to provide a total CCI score. In CCI score, sixteen kinds of different comorbidities were classified in different categories. Participants with the comorbidity was calculated weighted 1, such as myocardial infarction, congestive heart failure, peripheral vascular disease, cerebrovascular accident, dementia, chronic obstructive pulmonary disease (COPD), connective tissue disease, peptic ulcer disease, mild liver disease and diabetes mellitus. Moderate to severe chronic kidney disease, hemiplegia, diabetes with end-organ damage, leukemia, tumor of any type and malignant lymphoma were weighted 2. Participants with moderate to severe liver disease were weighted 3. Acquired immune deficiency syndrome (AIDS) and metastatic solid tumor were weighted 6 [9].

### Statistical Analysis

Data analysis compared distributions of demographic variables and the CCI score between the RA and comparison groups. The chi-square test was used to determine the differences between the 2 groups regarding the distribution of categorical demographic variables. Mean ages and mean CCI scores between the 2 groups were examined using Student's t-test. Incidence rates of NTM disease and active PTB were calculated for both groups (per 10,000 person-years) stratified by demographic variables and CCI scores. The incidence density of outcome (NTM disease or active PTB) was calculated by using the number of incident NTM disease or active PTB dividing by person-years at risk in both groups. We used the Poisson regression model to test the different incidence density of outcome (NTM disease or active PTB) between RA and non-RA groups and present with the incidence rate ratio (IRR) and 95% confidence interval (95% CI). We also used multivariable Cox proportional hazards regression analysis to measure the adjusted hazard ratio (aHR) of NTM disease and active PTB with 95% CI for the RA group compared with the comparison group, while manual controlling for sex, age, and CCI score. We assessed changes in the risk and aHR of NTM disease and active PTB during the follow-up years by using time-dependent covariates (<2 y, 2–4 y, 4–6 y, ≧6 y after RA diagnosis). The Kaplan-Meier method was used to plot the cumulative proportions of the studied subjects free of active PTB and NTM disease during the follow-up period, and the log-rank test was used to assess the differences between these curves.

All statistical analyses were performed using the SAS 9.1 statistical package (SAS Institute Inc., NC, USA). R software (R Foundation for Statistical Computing, Vienna, Austria) was used to prepare the Kaplan–Meier survival curves. A *P*<.05 in 2-tailed tests was considered significant.

## Results

### Characteristics of the Study Participants

In this study, we identified 29 131 patients for the RA group and selected 116 524 patients for the comparison group (Table 1). Both groups were similar in sex and age distribution and women (77.2%) and younger participants (78% <65 y of age) were dominant. The comorbidities were more prevalent in the RA group than non-RA group at the baseline (0.43±0.89 vs 019±0.67, *P*<.0001).

**Table 1:** Demographic status and Charlson comorbidity index score in cohorts with and without rheumatoid arthritis.

	Rheumatoid arthritis				
	No (N = 116524)		Yes (N = 29131)		
	n	%	n	%	*p*-value
Sex†					0.98
Women	89896	77.2	22474	77.2	
Men	26628	22.8	6657	22.8	
Age, year†					0.98
<50	48856	41.9	12214	41.9	
50–65	42052	36.1	10513	36.1	
≥65	25616	22.0	6404	22.0	
Mean (SD)#	52.3 (15.7)	52.4 (15.6)	0.4325		
Charlson comorbidity index, classification†					<.0001
None	104101	89.3	21111	72.5	
1–2	10010	8.6	6945	23.8	
≥3	2413	2.1	1075	3.7	
Mean of score (SD)#	0.19(0.67)		0.43(0.89)		<.0001

† Chi-square test;

# Two sample t-test.

### Incidence Rate Ratios of NTM Disease and active PTB

The incidence of NTM disease was 4.22 times greater in the RA group than in the non-RA group (1.91 vs 0.45 per 10,000 person-years), with an adjusted HR of 4.17 (95% CI = 2.61–6.65) (Table 2). However, the incidence of PTB was 2.99 times greater in the RA group than in the non-RA group (25.3 vs 8.46 per 10,000 person-years), with an adjusted HR of 2.87 (95% CI = 2.55–3.23). The risks of developing NTM disease and active PTB were greater for men than for women in both cohorts, although the incidence of both was much greater in the RA group. Patients with comorbidities had higher incidences of NTM disease and active PTB in both groups, rates of which were also higher in the RA group than in the non-RA group.

**Table 2:** Risks of non-TB mycobacterial disease and active pulmonary tuberculosis measure by sex, age and Charlson index score for study cohorts.

	Rheumatoid arthritis							
	No			Yes			Compared to non-Rheumatoid arthritis	
Variables	Event	PY	Rate^#^	Event	PY	Rate^#^	IRR* (95% CI)	Adjusted HR^†^ (95% CI)
**Non-TB mycobacterial disease**	36	796901	0.45	37	193934	1.91	4.22(4.05–4.40)***	4.17(2.61–6.65)***
Sex								
Women	18	623372	0.29	18	152377	1.18	4.09(3.90–4.29)***	3.80(1.95–7.38)***
Men	18	173530	1.04	19	41557	4.57	4.41(4.06–4.79)***	4.58(2.38–8.83)***
Age, years^†^								
<50	6	347795	0.17	7	87315	0.80	4.65(4.35–4.97)***	3.77(1.24–11.49)*
50–65	12	347795	0.41	16	70999	2.25	5.49(5.12–5.88)***	4.94(2.30–10.61)***
≥65	18	156982	1.15	14	35621	3.93	3.43(3.15–3.73)***	3.49(1.71–7.11)***
Charlson comorbidity index								
No	27	726318	0.37	18	143213	1.26	3.38(3.23–3.54)***	3.82(2.10–6.94)***
Yes	9	70585	1.28	19	50721	3.75	2.94(2.63–3.28)***	3.62(1.61–8.15)**
**Active Pulmonary Tuberculosis**	673	795227	8.46	487	192585	25.29	2.99(2.89–3.09)***	2.87(2.55–3.23)***
Sex								
Women	353	622385	5.67	269	151563	17.75	3.13(3.00–3.26)***	2.92(2.48–3.42)***
Men	320	172842	18.51	218	41022	53.14	2.87(2.68–3.07)***	2.81(2.36–3.35)***
Age, years^†^								
<50	74	347526	2.13	66	87068	7.58	3.56(3.36–3.77)***	2.95(2.10–4.14)***
50–65	164	291652	5.62	179	70451	25.41	4.52(4.27–4.79)***	3.89(3.13–4.83)***
≥65	435	156049	27.88	242	35065	69.01	2.48(2.31–2.65)***	2.26(1.93–2.65)***
Charlson comorbidity index								
No	406	725228	5.60	243	142548	17.05	3.05(2.93–3.17)***	3.43(2.93–4.03)***
Yes	267	69999	38.14	244	50037	48.76	1.28(1.18–1.39)***	1.82(1.52–2.17)***

Rate^#^, incidence rate, per 10,000 person-years; IRR^*^, incidence rate ratio; Adjusted HR^†^: model manual adjusted for sex, age, and CCI score; p<0.05, **p<0.01, ***p<0.001.

### Trend in Follow-up Times

Table 3 shows the incidence of NTM disease and active PTB according to follow-up time. In the first 2 years of follow-up, the RA group had a higher incidence than the non-RA group for both NTM disease (2.10 vs 0.39 per 10,000 person-years) and PTB (29.5 vs 8.02 per 10,000 person-years). The corresponding adjusted HRs were 4.98 and 3.39 for the RA group compared with the non-RA group.

**Table 3:** Trends of non-TB mycobacterial disease and active pulmonary tuberculosis risk by follow-up years.

	Rheumatoid arthritis							
	No			Yes			Compared to non-Rheumatoid arthritis	
Follow time, years	Event	PY	Rate^#^	Event	PY	Rate^#^	IRR^*^ (95% CI)	Adjusted HR^†^ (95% CI)
**Non-TB mycobacterial disease**								
<2	9	229578	0.39	12	57145	2.10	5.36(5.12–5.60)***	4.98(2.08–11.92)***
2–4	7	202095	0.35	6	49851	1.20	3.47(3.32–3.64)***	3.55(1.17–10.74)*
4–6	4	155564	0.26	7	37788	1.85	7.20(6.82–7.61)***	6.75(1.94–23.55)**
≥6	16	209665	0.76	12	49150	2.44	3.20(3.02–3.39)***	3.34(1.55–7.21)**
**Active Pulmonary Tuberculosis**								
<2	184	229455	8.02	168	57008	29.47	3.68(3.54–3.82)***	3.39(2.74–4.18)***
2–4	177	201765	8.77	113	49598	22.78	2.60(2.50–2.70)***	2.53(1.99–3.21)***
4–6	153	155132	9.86	99	37466	26.42	2.70(2.58–2.82)***	2.59(2.00–3.35)***
≥6	159	208874	7.61	107	48514	22.06	2.90(2.76–3.05)***	2.89(2.25–3.72)***

Rate^#^, incidence rate, per 10,000 person-years; IRR^*^, incidence rate ratio; Adjusted HR^†^: model manual adjusted for sex, age, and CCI score; *p<0.05, **p<0.01, ***p<0.001.

### Cumulative Incidence of NTM disease and active PTB Between the RA Group and Non-RA Group

**Figure 1:**
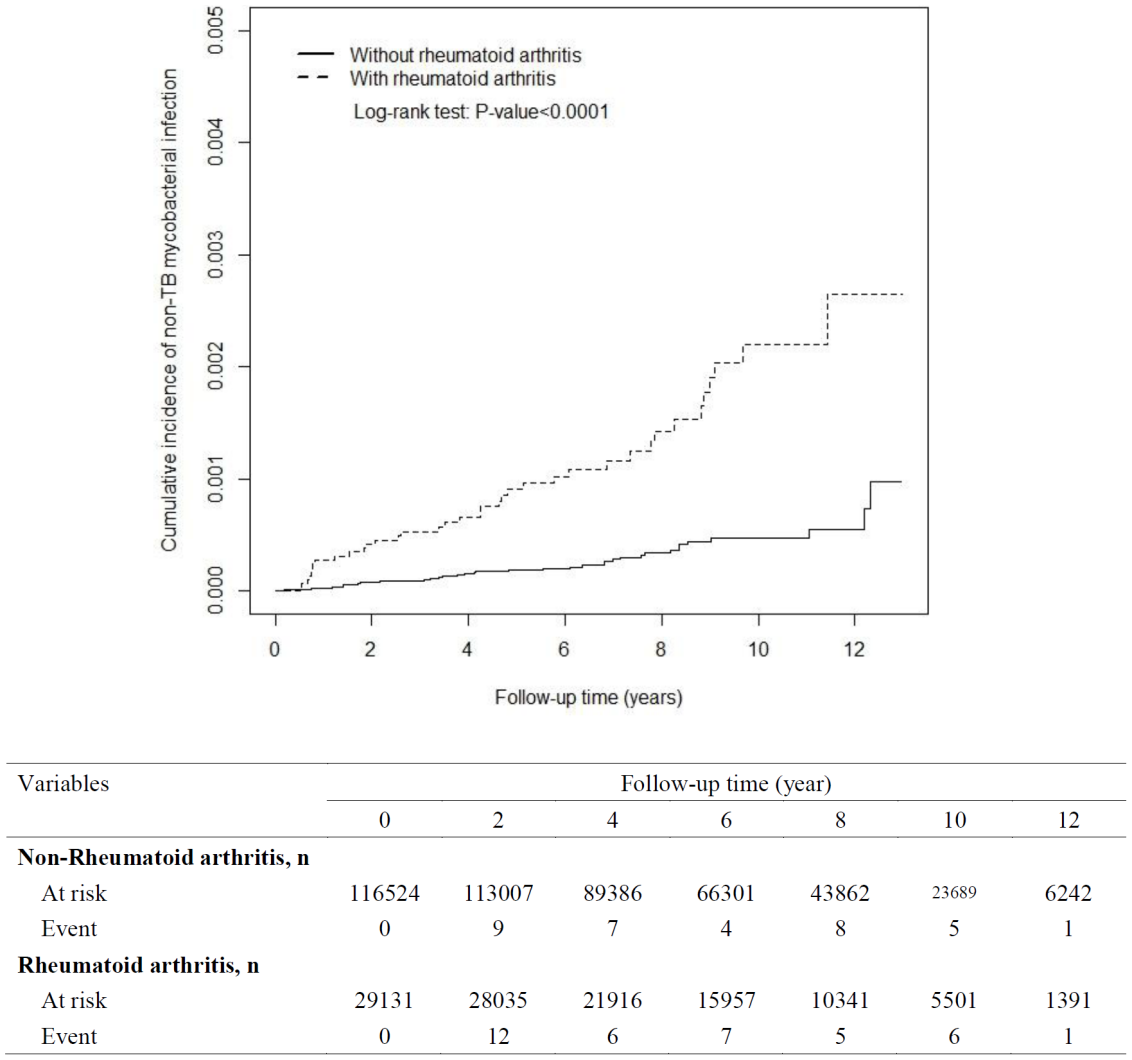
Cumulative incidence of non-TB mycobacterial disease for patients with rheumatoid arthritis (dashed line) and comparison subjects (solid line).

**Figure 2:**
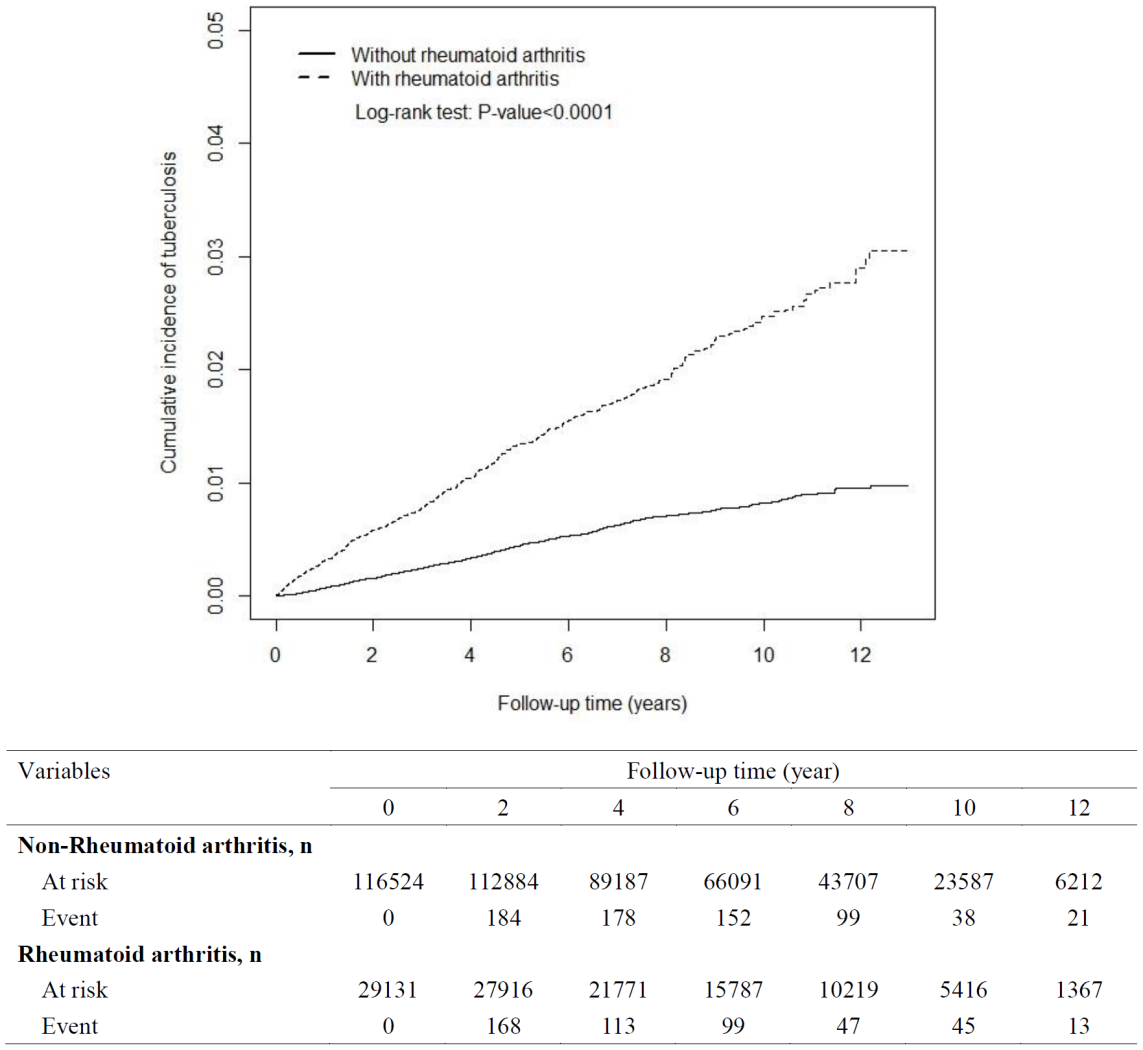
Cumulative incidence of active pulmonary tuberculosis for patients with rheumatoid arthritis (dashed line) and comparison subjects (solid line).

With 13 years of follow-up, Kaplan-Meier analysis showed that the cumulative incidence of NTM disease was 0.175% higher in the RA group than in the non-RA group. The incident number declined steadily in the RA group. The cumulative incidence of active PTB was 2.0% greater in the RA group than in the non-RA group. However, the event number declined faster in the RA group than in the non-RA group (Figures 1 and 2).

## Discussion

The first crucial result of our study as those RA patients' risks of developing NTM disease and active PTB were increased 4.17-fold and 2.87-fold compared with the general population. The age-stratified effects of the RA cohort on NTM disease and active PTB development were high in patients between 50–65 years of age (yielding HRs of 4.94 and 3.89, respectively) and >65 years of age (yielding HRs of 3.49 and 2.26, respectively). The risks of developing NTM disease and active PTB increased with age in both groups with a much greater gradient in the RA group. Our results were similar to those of Winthrop et al, who observed that among anti-TNF users (n = 8418, 60% with RA; 18 with NTM disease, 12/18 with pulmonary NTM; 16 with active TB, 11/16 with active PTB), NTM case-patients with a median age of 68 years active TB case-patients with a median age of 57.5 years were both more likely to have RA [21]. Our study concurs with the results of numerous other reports regarding the frequency among older people (>50 years) of mycobacterial infections such as NTM disease [22] and active PTB [23].

The second crucial result is that the risk of NTM disease significantly increased in the first 2 years (rate = 2.10) and ≧6 years (rate = 2.44) of RA diagnosis, whereas the rate of active PTB was higher in the first 2 years of diagnosis (rate = 29.47). Winthrop et al found that, among anti-TNF users (60% with RA), active TB case-patients exhibited a median time to onset after drug start of 670 days (<2 y), which was similar to that indicated in our study. The NTM case-patients exhibited a median time to onset after drug start of 1024 days. This result slightly differed with that in our study [21]. In our study, the risk of NTM significantly increased in the first 2 years and ≧6 years of RA diagnosis. After adjusting for HRs, both NTM disease (HR: 4.98) and PTB (HR: 3.39) in the first 2 years of follow-up had higher incidents in the RA group compared with the non-RA group. NTM disease (HR: 3.34) during ≧6 years of RA diagnosis was also more prevalent in the RA group than in the non-RA group. In a previous study, active PTB was found to be a predisposing factor [24] for the development of pulmonary NTM disease, which might explain this discrepancy. Additionally, the risk of developing NTM disease progressively increased with follow-up period; this may be associated with RA [25] and the aging process [26].

Disorders of the immune system can result in autoimmune diseases, inflammatory diseases, and cancer [27]. In certain instances, a primary immunodeficiency disease (PIDD) is associated with autoimmune disorders such as rheumatoid arthritis [28]. Having both cellular and antibody deficiencies carries an increased risk for juvenile RA [29]. RA increases the risk of infections [13]. Whether opportunistic infections resulting from mycobacteria occur more frequently in patients with RA, independent of immunosuppressive therapy, is uncertain [13], [14], [15]. Previous studies have proposed that an altered lymphocyte homeostasis in RA that may contribute to the autoimmune response as well as to the immunodeficiency in these patients [30].

A relationship between active TB and RA is currently recognized, and is primarily attributable to the immunosuppressive therapies used to treat RA. TNF is essential for controlling mycobacterium tuberculosis infection and cannot be replaced by other proinflammatory cytokines. The overproduction of TNF may cause immunopathology, whereas defective TNF production results in uncontrolled infection. The critical role of TNF in controlling tuberculosis has been recently illustrated by primary reactivation of latent infection in certain patients under pharmacological anti-TNF therapy for RA [31]. Therefore, RA patients treated with TNF-alpha inhibitors exhibit an increased incidence of active TB [32] compared with RA patients who are not being treated with TNF-alpha inhibitors [15]. NTM disease is also frequently found in patients being treated for RA by using anti-TNF therapy [21]. Whereas active TB can complicate the successful management of RA, NTM disease has perhaps become as challenging as (if not more so than) active TB in the RA setting, and represents an even greater challenge to rheumatologists that wish to use immunosuppressive therapies [33]. In the RA cohort without CCI displayed a 3.82-fold higher risk of developing NTM disease and the 3.43-fold higher risk of developing PTB compared with the comparison cohort (all *P*<.05) supported that RA cohort play a critical role for development of NTM disease and active PTB even without CCI (Table 2). Meanwhile Seong et al. study, active PTB event in RA cohort without immunosuppressive therapies imply that RA cohort itself is a risk factor of mycobactereial infection [14].

In this study, we found that CCI score was a strong predictor of the increased risk of both NTM disease (HR: 2.87) and active PTB (HR: 1.82) in the RA cohort (Table 2). The CCI score in the RA cohort was also higher than that in the non-RA cohort (0.19±0.67 vs 0.43±0.89, *P*<.001) (Table 1). In Crowson et al, RA disease characteristics and comorbidities were used to accurately assess the risk of serious infection in patients with RA in accordance with this finding [2]. Therefore, providing adequate RA care based on CCI scores is a crucial step in preventing the further development of NTM disease and active PTB [12]. Thus, a multidisciplinary team should guide the assessment, treatment, and holistic care of RA patients [34].

The greatest risk of developing NTM disease and active PTB was observed within 2 years of RA diagnosis. This phenomenon may be associated with RA identification at a later stage, strictly based on the 1987 American College of Rheumatology (ACR) diagnostic criteria [16] used in Taiwan. The ACR and European League Against Rheumatism (EULAR) posited revised classification criteria to focus on features at earlier stages of RA in 2010 [35], which may be helpful in identifying patients who may benefit from early effective intervention. Preventing active PTB development may also reduce the risk of NTM disease [24].

The primary (80%–85%) NTM disease is pulmonary NTM disease [36], [37], which may develop into acute respiratory failure (ARF) and burden hospitals [38]. Active PTB disease also presents as ARF and admission to the intensive care unit [39]. We suggest that RA is a critical factor for developing NTM disease and active PTB, irrespective of CCI score. Among RA patients, the therapeutic outcomes of pulmonary diseases caused by NTM diseases in a biological therapy setting were favorable [40].

With 13 years of follow-up, Kaplan-Meier analysis showed that the cumulative incidence of NTM disease and active PTB were higher in the RA group than in the non-RA group (0.175% for NTM disease, 2.0% for active PTB). The incidence of NTM disease and active PTB declined in the RA group. Therefore, it is necessary for physicians to detect NTM disease and active PTB patients at any stage of RA diagnosis among the clinical course. This study can serve to increase awareness of clinical physicians of the necessity of the early detect of pulmonary NTM disease and active PTB in RA patients, even those without CCI scores that are cause for concern. Because anti-RA therapy as this factor was not controlled in the study, therefore anyone on anti-TNF therapy will show increased NTM and PTB regardless of their RA status also.

Finally, bias in cohort studies include: diagnosis bias and loss to follow-up [41]. In this study, the cohort based on Registry for Catastrophic Illness Patient Database (RCIPD) and in-hospital patients. Meanwhile, the healthcare system of diagnosis of NTM disease and active PTB is relatively well established as compared to that in previous reported areas [43]. These policies avoid the diagnostic bias. Furthermore, we assess changes in the risk and adjusted hazard ratio of NTM disease and active PTB disease during the follow-up years by using time-dependent covariates for avoiding the follow-up bias.

### Limitations

Several limitations must be considered when interpreting these findings. The NHIRD does not provide detailed lifestyle information, such as information regarding smoking, body mass index, or physical activity, all of which are potential confounding factors in this study. However, RA patients undergo RA treatment and must modify their lifestyles; these may be implicated as factors for pulmonary infection in RA patients. Additionally, information on the RA severity scale, such as disease activity, functional impairment, and physical damage was unavailable in our data. The lack of drug data, such as data regarding treatment with anti-TNF drugs, MTX, and glucocorticosteroids, could be another limitation, as treatment with these drugs was not adjusted for in determining the outcomes under study. The incidence of NTM disease and active PTB in RA cohorts that are and are not receiving drug treatment requires further comparison. Despite the meticulous study design, which was employed to control for confounding factors, a key limitation of this study was its potential for bias caused by possible unmeasured or unknown confounders.

### Strengths

The strength of this study is that it is a nationwide population-based cohort longitudinal analysis of the risk of NTM disease and active PTB development in Asian people with RA. This cohort study is representative of the general population [42], and our findings can be generalized to the general population.

## Conclusion

This study raises awareness among clinical physicians regarding the necessity of the detection of NTM disease and active PTB in RA patients among the any stage of the clinical course even without CCI.
